# The infected blood inquiry: Impact on public perceptions of blood supply risk, safety, and donation attitudes

**DOI:** 10.1111/tme.13108

**Published:** 2024-11-12

**Authors:** Richard Mills, Eva‐Maria Merz, Mark Croucher, Barbara Masser, Susan R. Brailsford, Robert Smith, Eamonn Ferguson

**Affiliations:** ^1^ School of Psychology The University of Nottingham Nottinghamshire UK; ^2^ National Institute for Health and Care Research Blood and Transplant Research Unit in Donor Health and Behaviour, Department of Public Health and Primary Care University of Cambridge Cambridge UK; ^3^ Department of Sociology Vrije Universiteit Amsterdam Amsterdam The Netherlands; ^4^ Department of Donor Medicine Research Research Group on Donor Studies, Sanquin Research Amsterdam The Netherlands; ^5^ Donor Experience Services NHS Blood and Transplant, UK; ^6^ Research and Development Australian Red Cross Lifeblood Brisbane Australia; ^7^ School of Psychology The University of Queensland Brisbane Queensland Australia; ^8^ Microbiology Services, Colindale Blood Centre NHS Blood and Transplant London UK

**Keywords:** blood safety, donor attitudes, infected blood inquiry, perceived risk

## Abstract

**Background:**

The UK's Infected Blood Inquiry (IBI) highlighted a major public health scandal, with at least 30 000 people infected and more than 3000 deaths attributable to infected blood and blood products. This study investigates the impact of the IBI announcement on May 20, 2024, on public perceptions of blood supply risk, safety, and donation intentions in the UK compared to the USA.

**Methods:**

A 2 (country: UK vs. USA) × 2 (time: pre‐, post‐IBI announcement) between‐within‐subject study was conducted with 1635 participants (888 UK, 747 USA). Pre‐IBI data were collected from May 3 to 7, 2024, and post‐IBI data from May 30 to June 30, 2024. Key measures were perceived infection risk from transfusion, transfusion safety, willingness to donate and encourage others. The impact was assessed using differences‐in‐differences (DiD) and reliable‐change‐indices (RCI).

**Results:**

UK participants showed a significant but small decrease in perceived safety compared to USA participants, with 1 in 30 UK individuals perceiving a significant reduction in perceived transfusion safety. Decreases in perceived safety were associated with significant decreases in willingness to donate and encouragement of others in the whole sample and in USA participants and significant decreases in willingness to encourage others in UK participants. Older people reported a greater reduction in safety, and non‐donors were more likely to be put off donating and not ask others to donate as a result of their perception that safety had been reduced.

**Conclusion:**

Overall, perceived safety decreased marginally in the UK general population. Future research should explore the long‐term impacts of the IBI.

## 1 INTRODUCTION

The use of infected blood and blood products, examined in the Infected Blood Inquiry (IBI), highlights one of the most severe public health scandals in the UK's history.^1^, ^2^ The IBI showed that between 1970 and the early 1990s, over 30 000 NHS patients received blood transfusions, or blood products, infected with hepatitis C or HIV. Of these, around 3000 have since tragically died, severely impacting their families and loved ones.^1^, ^2^ These infections were primarily due to the UK importing clotting factors, and other blood products from paid donors overseas as it was unable to meet domestic demand.^1^, ^3^ These plasma products were often manufactured from large pools of donors (up to 60 000) that included donors from high‐risk populations, meaning that one infected donation could contaminate an entire batch.^1^, ^4^, ^5^ In relation to whole blood donation, the IBI saw that some donor selection measures in the UK took too long to implement (e.g., the ‘AIDS leaflet’), that additional tests for hepatitis C could have been introduced sooner, and there were delays in decision making not helped by the regional nature of services at this time (page 5, vol 1).^1^

Persistent efforts by the affected individuals and advocacy groups eventually led to the establishment of an independent public statutory inquiry.^1^ Led by Sir Brian Langstaff, a former High Court judge, and his team, the inquiry commenced on July 11th 2017 and was tasked with investigating the circumstances that led to the use of infected blood and blood products in the UK. Over several years, the IBI collected extensive evidence from numerous groups—including victims (the infected), families (the affected) and countless other stakeholders such as expert groups, medical professionals, and government witnesses (see timelines and groups involved here). The findings of the IBI, released in seven volumes on May 20th, 2024 (https://www.infectedbloodinquiry.org.uk/), confirmed the failure to act and the inability of successive governments to properly investigate the scandal. In response, the UK Prime Minister at the time, Rishi Sunak^6^ issued a public apology—‘a day of shame for the British state’—acknowledging the government's failure and promising compensation for the ‘infected’ and ‘affected’.^7^

This research explores the short‐term impact of the announcement of the IBI findings on the general public's perceptions regarding overall blood safety and donation behaviour (i.e., people's willingness to donate blood and encourage others to donate). We examine perceptions of the safety of blood as an index of people's perceptions of the overall safety of processes linked to blood transfusion and the use of other blood products. That is, people are unlikely to be aware of the subtle difference between types of blood products and whole blood versus plasma. Thus, their overall perception of the safety of blood for transfusion acts as a good general index of the safety of blood products within the general population.

People perceive risk as higher and safety as lower when the risk could potentially affect them.^8^, ^9^, ^10^ Furthermore, people judge risk in terms of potential consequences (the harm that they may experience) rather than by probabilities,^8^, ^11^ and extract gist information about risk based on heuristics such as the availability heuristic.^9^, ^12^ Therefore, we hypothesise that compared to the USA, people in the UK will show reductions in their perceptions of overall blood safety and increased perceptions of infection risk from transfusion. Furthermore, we would expect that people may wish to psychologically and behaviourally distance themselves from an action perceived as harmful to others.^13^ This may result in a generally reduced willingness to be a blood donor and willingness to encourage others to donate as well.

Given the historical nature of the events comprising the IBI and the literature on risk, we also explore whether these effects vary by various (socio‐)demographics that are comparable across the two countries in terms of age, sex, education, and donor status. This research provides valuable insights into the short‐term impacts of the IBI on public perceptions and behaviour of the overall supply of blood and blood products, underscoring the importance of maintaining transparency and accountability in public health communications and practices.

## 2 MATERIALS AND METHODS

### 2.1 The sample

The sample consists of 1635 adults from the general populations in the USA (*N* = 747) and the UK (*N* = 888), from which we collect data pre‐IBI (between 3rd and 7th May 2024) and post‐IBI (between 30th May and 30th June 2024). The data were collected through an online platform Prolific, with the survey constructed on Qualtrics, quota balanced across gender (female, male). Participants were paid around £9 ($11) p/h for each survey (payment information). Prolific has a built‐in option for follow‐up studies (link). The initial survey was part of a larger study surrounding perceptions of incentives in whole blood (See Supplementary ‘Prolific survey questions’ File for more information). Due to the high sensitivity of the IBI, especially among those who have received transfusions, and our focus on willingness to donate (which requires eligibility to donate), only non‐recipients of blood were recruited for the post‐IBI study.

We employ causal econometric techniques to quantify the effect of the IBI announcement on the general population, as well as on specific demographic groups (e.g., age, sex, and previous donor history). The rationale for including the USA as a comparison country is that it is likely to have received comparatively less media coverage surrounding the IBI than the UK. As a result, we would expect comparable responses across the two time periods with respect to perceptions of risk and safety of the blood supply in the USA (i.e., little variation), but negative effects on perceptions of risk and safety in the UK. Additionally, the USA is demographically and culturally similar to the UK, with a comparable blood donation system. Other European countries, though arguably similar to the UK, would have likely received more media coverage surrounding the IBI and are, thus, less suitable comparators.

### 2.2 Design and timelines

#### 2.2.1 Design

We conducted a 2 (Country: UK, USA) by 2 (Time: pre‐post) between‐within‐subject study. The between‐subject factor was the country, with the USA as a control for change in the UK (see statistical analysis section below). The within‐subject factor was the pre‐post IBI data collection. The majority of participants (93.8%) answered the post‐IBI survey in the first week (i.e., between 30th May and 5th June 2024) (Figure S1 in the Supplementary File).

### 2.3 Measures

The following were assessed in both the pre‐ and post‐IBI surveys:

**Infection risk and transfusion safety**: *Perceived infection risk* was measured by: ‘*What do you feel the level of infection risk is to a patient receiving blood in the UK/USA?*’ (country varied by country of current residence) (from 1 = ‘No risk at all to 5 “An extremely large risk”’). *Perceived transfusion safety* was assessed by: *‘To what extent do you feel it is safe in the UK/USA to have a blood transfusion if you need one?’* (from 1 = ‘Not at all safe’ to 11 ‘Completely safe’).

**Willingness to donate and encourage others**: Willingness to donate (*‘I am willing to donate blood (assuming you are eligible)’* and willingness to encourage others (‘*I am willing to encourage others to donate blood’*) were self‐reported on 1= ‘Not at all’ to 7 = ‘Completely’ scales. These measures correlate with donation behaviour while also measuring two distinctions: (i) willingness to donate is approaching (Approach) the decision to donate, and (ii) encouraging others is about thinking of the wider social decision‐making of others to donate (Encourage).^14^, ^15^

**Demographics**: We collected data on age, sex, education and donation history. Age was measured in years and split into three categories: (i) ‘Gen Z’ (18–26 years) (=1), (ii) Millennials (‘27–42’ years) (=2), and (iii) ‘Gen X + Boomers’ (>42 years) (=3). These categorisations were chosen because these generational groups are widely recognised and understood, allowing for more practical insights into groups that might be affected.^16^ Sex was assessed as female (=1) and male (=0), with the option of prefer not to say, coded as missing (=.). Education was defined as either non‐tertiary (=0) or tertiary (=1) educated. For blood donor history, we distinguish between those who have (i) never donated blood (=1) and those who have donated blood at least once (=2). These demographic questions were only asked in the initial pre‐IBI survey (given the relatively short period between the two data collection points).

We also asked several other questions about ethnicity, income, political ideology, and region (country of residence and region/state currently residing). Given differences across countries (USA/UK), the questions differed slightly (i.e., income brackets and ethnicity are defined differently in the USA relative to the UK). As such, we focus on age, sex, education, and donor history, which are characteristics where response options are comparable across the two contexts.

### 2.4 Timelines and cultural context

Generally, it has been recommended that some cultural context should be provided to aid in understanding research findings.^17^ To this end, we explored the media coverage of the IBI in the UK and USA, covering the pre‐ and post‐IBI data collection period. This provides a descriptive backdrop to the broader penetration of the IBI announcement. A web search targeted the top four news websites in the USA,^18^ and the BBC in the UK. The search was conducted through the news website search function and supplemented with ‘Google Site Search’ through the search term ‘infected blood’. The timeframe aimed to capture pre‐during‐post announcement of the IBI, covering April 2024–June 2024.

In addition, we utilised Google Trends data to gauge public interest in the term ‘Infected Blood’ across the UK and USA during the same period. Google's ‘Interest’ index measures ‘…search interest relative to the highest point on the chart for the given region and time. A value of 100 is the peak popularity for the term. A value of 50 means that the term is half as popular. A score of 0 means there was not enough data for this term’.

This combined approach provides insights surrounding media coverage and public search behaviour, offering a clearer picture of how the IBI was represented and received by the public in the two countries.

### 2.5 Statistical analysis and power calculations

Statistical analyses were conducted in Stata 18. All tests are two‐tailed. Statistical significance is determined by *p* < 0.05. We explore the effects of the IBI both at the aggregate population level using regression models but also at the level of each individual in the sample using the Reliable Change Index (RCI) proposed by Jacobson and Truax.^19^

#### 2.5.1 Causality—difference‐in‐difference (DiD) analysis

To examine causality, we ran a two‐period fixed effects DiD model. The DiD model allows us to infer a causal effect of the IBI announcement by comparing changes in outcomes before and after the announcement between individuals in the UK (treatment group) and the USA (control group).

The DiD model used to quantify the change in safety involved standardising both ‘infection risk’ (M=0, SD=1) and ‘safety of transfusion’ (M=0, SD=1). A two‐period fixed‐effects DiD model was employed with the following specification described in Equation (1):

(1) $$y_{\mathrm{it}}=\alpha_{i}+\beta_{1}\left(\text{Post}\_{\mathrm{IBI}}_{it}\times {\mathrm{UK}}_{i}\right)+\gamma_{i}+\lambda_{t}+\epsilon_{\mathrm{it}},$$

where $y_{\mathrm{it}}$ is the outcome variable (i.e., infection risk and safety of transfusion), $\alpha_{i}$ the intercept, $\beta_{1}\left(\text{Post}\_{\mathrm{IBI}}_{\mathrm{it}}\times {\mathrm{UK}}_{i}\right)$ captures the difference‐in‐differences estimate, $\gamma_{i}$ represents the individual (panel) fixed effects, $\lambda_{t}$ the time‐fixed effects and $\epsilon_{\mathrm{it}}$ is the error term. Standard errors are clustered at the individual level to account for repeated observations over time. Both ‘infection risk’ and ‘safety of transfusion’ are standardised (M = 0, SD = 1).

#### 2.5.2 Reliable Change Index (RCI) analyses

To examine significant changes at the individual level, we construct an RCI for each individual's infection risk and safety.^19^, ^20^, ^21^, ^22^, ^23^ The RCI represents the raw change score (i.e., post‐IBI – pre‐IBI) divided by the index of random error resulting from unreliability. If the absolute value of this RCI exceeds 1.96, this represents a significant change for that individual. The RCI sign gives the direction of the change. However, current evidence suggests that a threshold of 1.645, reflecting 90% confidence that reliable change has occurred, should be adopted as the original threshold is overly stringent.^23^ Therefore, we adopt this threshold in our analysis. The formula for the RCI is described in Equation (2):

(2) $$\mathrm{RC}=\frac{{\bar{X}}_{\text{Post}\;\mathrm{IBI}}-{\bar{X}}_{\mathrm{Pre}\;\mathrm{IBI}}}{S_{\text{Diff}}},$$

where ${\bar{X}}_{\text{Post}\;\mathrm{IBI}}$ (mean of variable post‐IBI), ${\bar{X}}_{\mathrm{Pre}\;\mathrm{IBI}}$ (mean of variable pre‐IBI), $s_{1}$ (standard deviation of variable pre‐IBI), and $S_{\text{Diff}}=\sqrt{2{\left(S_{E}\right)}^{2}}$. $S_{E}=s_{1}\sqrt{1-r_{\mathrm{xx}}}$, with $r_{\mathrm{xx}}$ representing the test‐retest reliability of the variable (measured by Pearson's correlation between Pre‐IBI and Post‐IBI) (see Jacobson and Truax^19^ for more details).

#### 2.5.3 Impact of reliable change on negative changes in approach and encourage

We assess the impact of significant changes in infection risk and safety on negative changes in willingness to donate blood and encourage others to donate. We employ several logistic models, a class of binary responses (see Wooldridge^24^) of the following form (Equation (3)):

(3) $$P\left(△y_{i}=1|△x_{i}\right)=F\left(\beta_{0}+{\beta_{1}{\mathrm{RCI}}_{\text{infection risk}}}_{i}+\beta_{2}\;{{\mathrm{RCI}}_{\text{safety}}}_{i}+X_{i}\;\beta +\epsilon_{i}\right),$$

where $△y_{i}$ is the difference in the outcome variable between pre‐ and post‐IBI for each individual, turned into a ‘negative’ binary response (i.e., takes on a value of 1 if a decreased change willingness to donate or encourage others to donate is observed. $\beta_{0}$ is the intercept. $\beta_{1}\;{\mathrm{RCI}}_{\text{infection risk}}$ and $\beta_{2}\;{\mathrm{RCI}}_{\text{safety}}$ capture the reliable change in infection risk and safety for each individual, respectively. $X_{i}\;\beta$ represents a vector of additional controls (age, sex, education, and prior donation status), and $\epsilon_{i}$ is the error term. *F* is a function that takes on values strictly within zero and one, such that $0<F\left(q\right)<1$, for all real numbers q. The logistic function $F\left(q\right)=\frac{{ℯ}^{q}}{1+{ℯ}^{q}}$ is the cumulative distribution function for the standard logistic distribution. The logistic model is estimated using maximum likelihood estimation (see Wooldridge^24^ for more details). Standard errors are clustered at the individual level to account for repeated observations over time. Coefficients are described in terms of Odds Ratios (OR).

#### 2.5.4 Power analysis

Based on effect sizes reported by Merz et al.^25^ to achieve a power of 0.80 with alpha of 0.05 for a 2 (between) by 2 (within) design to detect an interaction with a small effect size (Cohen's *D* = 0.105) requires 855 in USA (pre and post) and 855 in the UK (pre and post).^26^

## 3 RESULTS

### 3.1 Field quasi‐experiment

**Sample summary statistics:** The pre‐IBI sample comprises 2060 observations (*N* = 1032 USA, *N* = 1028 UK) (Table S1 Supplementary File). Of these 2060 observations, 175 (17.96%) in the USA and 107 (10.41%) in the UK stated they were prior blood recipients. For reasons described above, these recipients were excluded from the post‐IBI study. As a result, 1778 participants (857 USA, 921 UK), were invited to participate in the post‐IBI study. Of these, 747 responded in the USA (87.16% response rate), and 888 (96.41% response rate) in the UK. Overall, these are good response rates, with a typical rule of thumb being that <5% attrition leads to little bias and >20% poses serious threats to validity.^27^ Furthermore, comparing the pre‐ and post‐IBI demographics, we see that younger people, particularly in the USA, were more likely to ‘drop out’ of the study (*p* < 0.01). There were no other differences in demography (i.e., sex, education and prior donor status) (Table S2 Supplementary File). Summary statistics for the main variables are in Table S3 in the Supplementary File.

**Perceptions of risk and safety:** The two measures are significantly negatively correlated, showing that an increased perception of risk is associated with a lower perception of safety (pre‐IBI: r=−0.51,p<0.01, post‐IBI: r=−0.52,p<0.01) (Tables S4–S6, Supplementary File).

**FIGURE 1:**
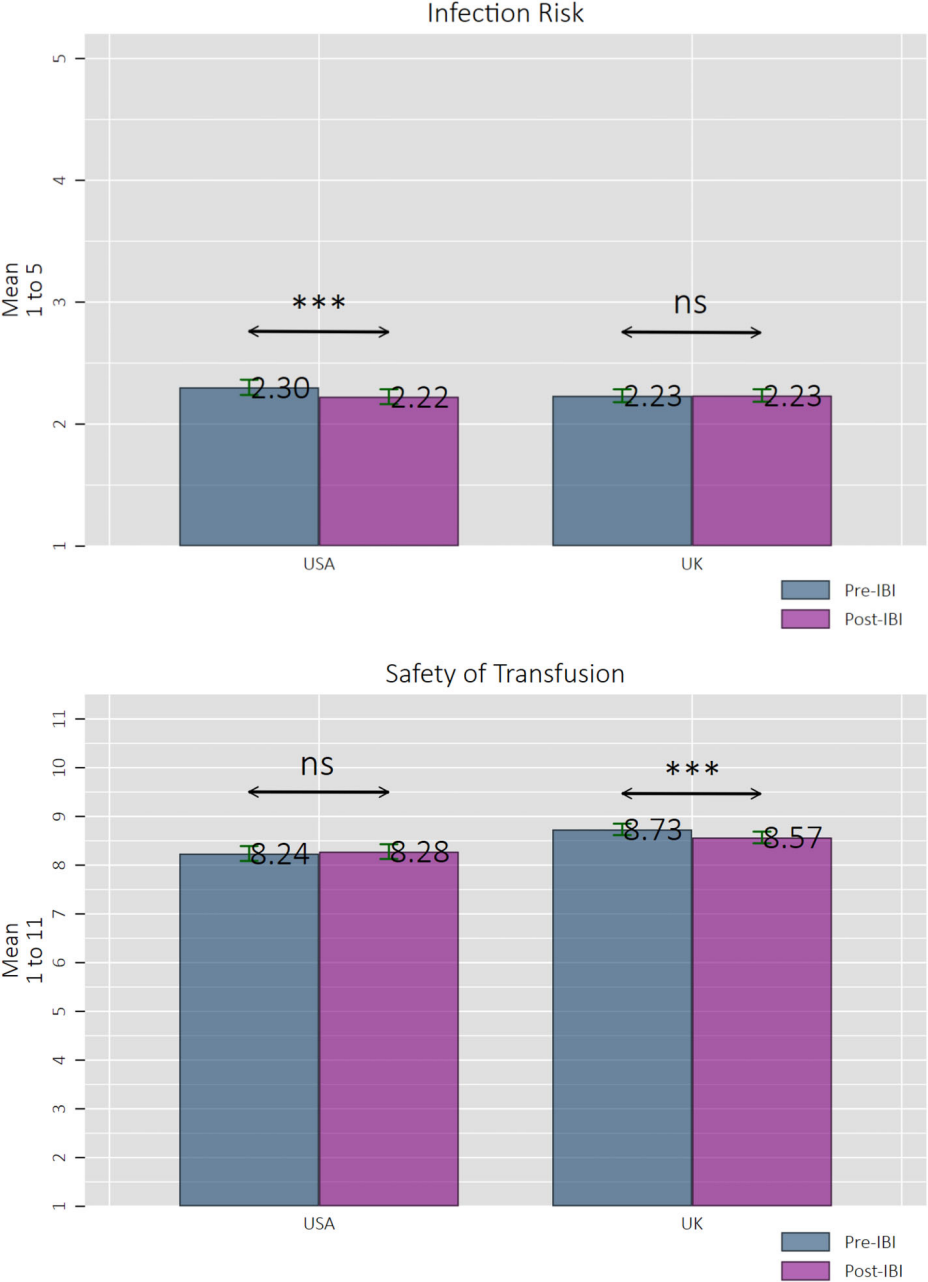
Perception of infection risk and transfusion safety. Perceived infection risk (‘What do you feel the level of infection risk is to a patient receiving blood in the UK/USA?’) (country varied by country of current residence) (from 1 = ‘No risk at all to 5 “An extremely large risk”’. Perceived transfusion safety (‘To what extent do you feel it is safe in the UK/USA to have a blood transfusion if you need one?’) (from 1 = ‘Not at all safe’ to 11 ‘Completely safe’). Confidence intervals (CIs) are 95%. ***p < 0.01; **p < 0.05; *p < 0.1.

Figure 1 presents the results for perceived (i) infection risk and (ii) safety of transfusion across country (UK/USA) and pre‐ and post‐IBI. Significance is determined by several ordinary least squares regressions (controlling for age, sex, education, prior donor status and region/states residing in), clustered at the individual level to account for repeated observations over time (Tables S7–S10 Supplementary File). For perceived infection risk, there was a significant (*p* < 0.01) decrease over time in the USA but no significant difference in the UK (*p* = 0.97).

For perceived safety of transfusion, there was no significant difference pre‐ and post‐ in the USA (*p* = 0.58), but a small but statistically significant decrease in the UK (*p* < 0.01). The decline in the UK was 1.83%, which is a small change. Furthermore, for both counties, we see that perceived infection risk is on the lower end of the scale, and perceived safety is on the higher end of the scale—reflecting low levels of ‘concern’ in the blood supply for both countries. Also, pooling across pre‐ and post‐IBI, the perceived safety of the blood supply is higher in the UK than in the USA (*p* < 0.01) (Table S3 in the Supplementary File).

Table 1 shows the DiD results, with the average treatment effect on the treated (ATET) being a significant increase in infection risk (*p* < 0.05) and a significant decrease in safety (*p* < 0.05). For perceived infection risk, this is due to the decrease in perceived infection risk in the USA sample, not an increase in the UK (as would have been expected). Regarding the magnitude of the effect on perceived safety, those in the UK perceived the blood supply to be 0.11 standard deviations less safe than those in the USA post‐IBI, which is a small effect.^26^, ^28^

**TABLE 1:** Two‐period fixed effects diff‐in‐diff models.

	(1)	(2)
Variables	Infection risk	Safety
ATET	**0.099** **	**−0.107** **
	(0.048)	(0.047)
Constant	0.021*	0.017
	(0.012)	(0.012)
Observations	3261	3261
# Observations	3261	3261
# Clusters	1635	1635

*Note*: Dependent variables are standardised (*M* = 0, SD = 1). ATET = Average Treatment Effect on the Treated, estimating the diff‐in‐diff with the UK and the treatment group and the USA as the control. A positive ATET indicates an increase in the dependent variable due to the treatment, while a negative ATET indicates a decrease. Cluster robust standard errors in parenthesis to account for repeated observations over time.

***p < 0.01;

** *p* < 0.05;

* *p* < 0.1.

These results show a small but significant decline in perceived safety in the UK sample. We observe no significant change in the level of perceived infection risk in UK participants. These results also do not vary across the responses of the ‘early’ completers (i.e., first week) and ‘late’ completers (after the first week).

### 3.2 Individual analysis (RCI)

**FIGURE 2:**
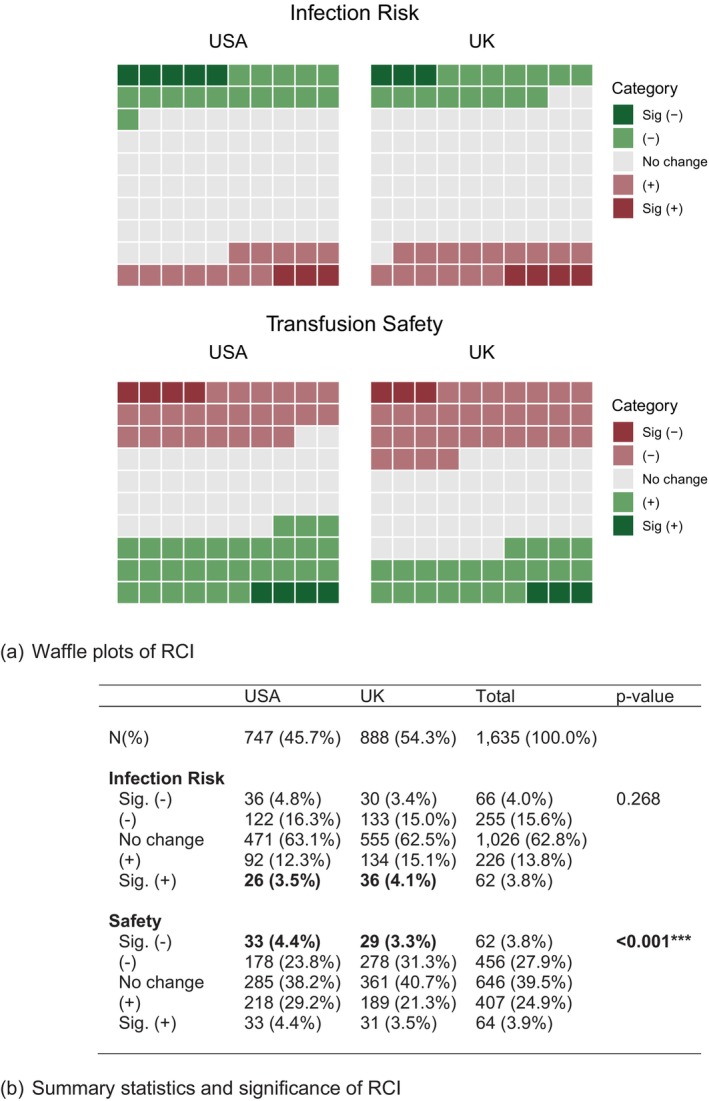
Reliable change indices (RCI) for risk and safety. (A) Waffle plots show relative percentages for RCI values of infection risk and safety. Each square represents a percentage (there are 100 squares in each figure). Note that the legend for infection risk is red for increases and green for decreases, while for safety it is red for decreases and green for increases; (B) Summary statistics of the RCI measures, with Chi‐squared tests (for proportions).***p < 0.01; **p < 0.05; *p < 0.1.

Figure 2 shows the reliable change (RCI estimates) from pre‐ to post‐IBI in infection risk and safety across the two countries (USA/UK) represented as Waffle plots. These represent the number of people who change (positively, negatively) per hundred and if this change is statistically significant for that individual. Looking at those who show a statistically significant change, we observe a significant increase in perceived risk for 4.1% of the UK sample and 3.5% of the USA sample (Figure 2). Overall, there were no significant differences between the two countries for this measure. For the UK sample, 3.3% had a significant drop in perceived safety and 4.4% in the USA sample—a difference that is not statistically significant (Figure 2). However, when looking at the overall distribution of the measure, we see that the UK sample is significantly different to the USA sample, primarily driven by the higher number of participants who had a drop in perceived safety overall (significant and non‐significant decreases) (Table S11 Supplementary File).

### 3.3 Impact of changes in perceived risk and safety

Table 2 shows the logistic regression results, expressed as odds ratios (OR), across the UK (columns 1–2), USA (columns 3–4) and for the total sample (columns 5–6). There were no significant effects on infection risk. The results show that a significant decrease in perceived transfusion safety, relative to no change, is associated with a significant decrease in willingness to donate in the USA (OR = 3.396, *p* < 0.01), and for the total sample (OR = 2.336, *p* < 0.01), but not in the UK (OR = 1.594, *p* = 0.31). For encouraging others to donate, a significant decrease in perceived safety is associated with a significant decrease in encouraging others in the UK (OR = 2.697, *p* < 0.05), USA (OR = 2.072, *p* < 0.05), and across the total sample (OR = 2.396, *p* < 0.01). Positive changes in perceptions of safety show no differences in reduced approach and encouraging others. Supplementary Tables S12 and S13 provide a robustness check using the simple change score and the sample restricted to one‐week post‐IBI.

**TABLE 2:** Logistic regressions (in odds ratios) for positive and negative changes across pre‐ and post‐IBI.

	UK		USA		Total	
Variables	↓ ∆ Approach	↓ ∆ Encourage	↓ ∆ Approach	↓ ∆ Encourage	↓ ∆ Approach	↓ ∆ Encourage
**Infection risk**: *Baseline: No change*						
Sig. (−)	0.675	1.118	0.728	0.903	0.706	0.962
	(0.350)	(0.497)	(0.352)	(0.415)	(0.243)	(0.298)
(−)	0.787	1.022	0.832	0.851	0.810	0.933
	(0.199)	(0.237)	(0.241)	(0.227)	(0.152)	(0.162)
(+)	0.825	0.656*	1.260	0.854	1.014	0.710*
	(0.208)	(0.153)	(0.374)	(0.265)	(0.187)	(0.131)
Sig. (+)	0.591	0.424*	1.671	1.089	0.956	0.654
	(0.286)	(0.190)	(0.825)	(0.526)	(0.308)	(0.205)
**Transfusion safety**: *Baseline: No change*						
Sig. (−)	1.594	**2.697** **	**3.396** ***	**2.072** **	**2.336** ***	**2.389** ***
	(0.730)	(1.135)	(1.363)	(0.754)	(0.692)	(0.655)
(−)	1.277	1.282	1.553*	1.531*	**1.381** **	**1.373** **
	(0.250)	(0.231)	(0.382)	(0.358)	(0.209)	(0.195)
(+)	1.278	0.889	0.869	1.166	1.039	0.984
	(0.294)	(0.193)	(0.220)	(0.278)	(0.176)	(0.155)
Sig. (+)	0.964	0.382*	1.413	0.712	1.156	0.534
	(0.472)	(0.214)	(0.673)	(0.390)	(0.386)	(0.209)
Constant	1.597	0.459	0.642	0.273*	0.890	0.270
	(1.088)	(0.368)	(0.469)	(0.213)	(0.588)	(0.217)
Region/State controls	✓	✓	✓	✓	✓	✓
Observations	880	880	705	714	1589	1598
Pseudo *R* ^2^	0.0422	0.0404	0.0648	0.0533	0.0407	0.0390
Log Likelihood	−458.6	−510.9	−364.2	−386.7	−834.1	−906.5
Degrees of Freedom	28	28	53	54	66	67
Chi^2^	38.80	42.40	54.55	47.12	69.97	72.79
Prob < chi^2^	0.0842	0.0397	0.415	0.735	0.346	0.293

*Note*: Logistic regressions (represented by odds ratios, OR) for decreased changes in approach (↓ ∆ Approach) and encouraging others (↓ ∆ Encourage) by country (UK, USA). Independent variables represent RCI values for (i) Infection Risk, and (ii) Transfusion Safety. Additional controls include age, sex, education, prior donor status and region/states residing in), clustered at the individual level to account for repeated observations over time. Cluster robust standard errors in parenthesis.

*** *p* < 0.01;

** *p* < 0.05;

* *p* < 0.1.

These results show that significant deviations in perceived transfusion safety are associated with decreased willingness to donate and encourage others to donate in the USA and the total sample. For the UK, we only see a significant association for encouraging others.

### 3.4 Exploratory analysis across age, sex and donor status

We explored the differences in increased perceptions of risk (↑∆ Risk), decreased perceptions of safety (↓∆Safety), decreased perceptions of approach (↓∆ Approach) and encouraging others (↓∆ Encourage) across age, sex, education and previous donor history. The results, summarised in Table 3, are derived from several logistic regressions. Below, we discuss the significant findings; non‐significant results are not reported (see Table S14, Supplementary file for regressions restricted to first‐week post‐IBI).

**TABLE 3:** Logistic regressions (in odds ratios) for exploratory demographics analysis.

	↑ ∆ Risk		↓ ∆ Safety		↓ ∆ Approach		↓ ∆ Encourage	
Variables	USA	UK	USA	UK	USA	UK	USA	UK
**Age**								
*Baseline: Gen Z*								
Millennials	**0.435** **	0.745	0.997	**2.248** ***	0.628	0.689	0.877	0.886
	(0.159)	(0.231)	(0.346)	(0.706)	(0.225)	(0.196)	(0.319)	(0.237)
Gen X + Boomers	**0.412** **	0.950	1.063	**2.417** ***	0.814	0.619*	1.189	0.801
	(0.150)	(0.295)	(0.372)	(0.769)	(0.288)	(0.180)	(0.426)	(0.216)
**Sex**								
*Baseline: Males*								
Female	0.861	**1.642** ***	0.966	1.237	0.760	1.045	0.927	0.767*
	(0.186)	(0.295)	(0.171)	(0.179)	(0.141)	(0.171)	(0.168)	(0.116)
**Education**								
*Baseline: Non‐tertiary*								
Tertiary	0.798	1.115	1.108	1.041	0.821	1.068	**1.600** **	1.243
	(0.181)	(0.214)	(0.215)	(0.161)	(0.165)	(0.187)	(0.322)	(0.199)
**Donor history**								
*Baseline: Non‐donors*								
Donor	1.196	0.780	0.872	0.812	0.890	**0.659** **	1.069	**0.665** **
	(0.269)	(0.144)	(0.154)	(0.122)	(0.170)	(0.116)	(0.200)	(0.106)
Constant	0.175	0.152***	0.171**	0.282***	0.794	0.734	0.207**	0.685
	(0.196)	(0.064)	(0.140)	(0.109)	(0.549)	(0.261)	(0.150)	(0.234)
Region/State controls	✓	✓	✓	✓	✓	✓	✓	✓
Observations	681	880	717	880	709	880	718	880
Pseudo *R* ^2^	0.0580	0.0248	0.0345	0.0236	0.0370	0.0341	0.0387	0.0188
Log Likelihood	−291.3	−417	−414.3	−554.5	−376.1	−462.6	−393.8	−522.4
Degrees of Freedom	36	16	42	16	42	16	43	16
Chi^2^	35.41	21.95	27.47	24.48	27.67	29.63	32.79	18.81
Prob < chi^2^	0.496	0.145	0.959	0.0795	0.957	0.0200	0.871	0.279

*Note*: Logistic regressions (represented by odds ratios, OR) for increased changes in risk (↑ ∆ Risk), decreased changes in safety (↓ ∆ Safety), decreased changes in approach (↓ ∆ Approach) and encouraging others (↓ ∆ Encourage). Key demographics of interest are age (Gen Z, Millennials, Gen X + Boomers), sex (male, female), education (non‐tertiary, tertiary), and prior donor status (non‐donor, donor). Additional controls include regions/states residing in. Standard errors are clustered at the individual level to account for repeated observations over time. Cluster robust standard errors in parenthesis.

*** *p* < 0.01;

** *p* < 0.05;

* *p* < 0.1.

#### 3.4.1 Age

Older participants: Millennials (OR = 0.435, *p* < 0.05) and Gen X + Boomers (OR = 0.412, *p* < 0.05) relative to Gen Z in the USA have a lower likelihood of having an increased change in their perception of infection risk. For the UK, Millennials (OR = 2.248, *p* < 0.05) and Gen X + Boomers (OR = 2.417, *p* < 0.01) relative to Gen Z are associated with a significantly higher likelihood of having decreased changes in the perception of safety. The latter results in the UK highlight that older people might be more affected by the announcement of the IBI report as they perceive themselves more at risk, in line with previous literature showing that younger people generally perceive themselves as less vulnerable to health risks.^29^, ^30^, ^31^ An alternative account is that they are psychologically closer to the inquiry, given the time when infected blood was around and their age, making them more aware of the historical context.^32^, ^33^

#### 3.4.2 Sex

Women in the UK had a significantly higher increase in perception of infection risk (OR = 1.642, *p* < 0.01). These results are in line with previous literature showing that women are generally more risk‐averse than men,^34^, ^35^, ^36^, ^37^, ^38^, ^39^ are more likely to perceive the same events as riskier than men^36^ and are more likely to require blood transfusions.^40^, ^41^, ^42^, ^43^, ^44^, ^45^

#### 3.4.3 Education

Education was generally not significantly associated with any of the outcome measures, except for reduced encourage in the USA. This may highlight that more educated people in the USA are more likely to follow the news.^46^

#### 3.4.4 Donor history

Blood donors in the UK are associated with a lower likelihood of having a decrease in approach (OR = 0.659, *p* < 0.05) and encourage (OR = 0.665, *p* < 0.01). These results are in line with the idea that blood donors are less affected by the announcement of the IBI than non‐donors, perhaps due to the continued belief that they are benefitting the well‐being of others,^47^, ^48^ and having higher trust in the NHS and NHSBT.^17^

### 3.5 Cultural context (UK and US media coverage of the IBI)

**FIGURE 3:**
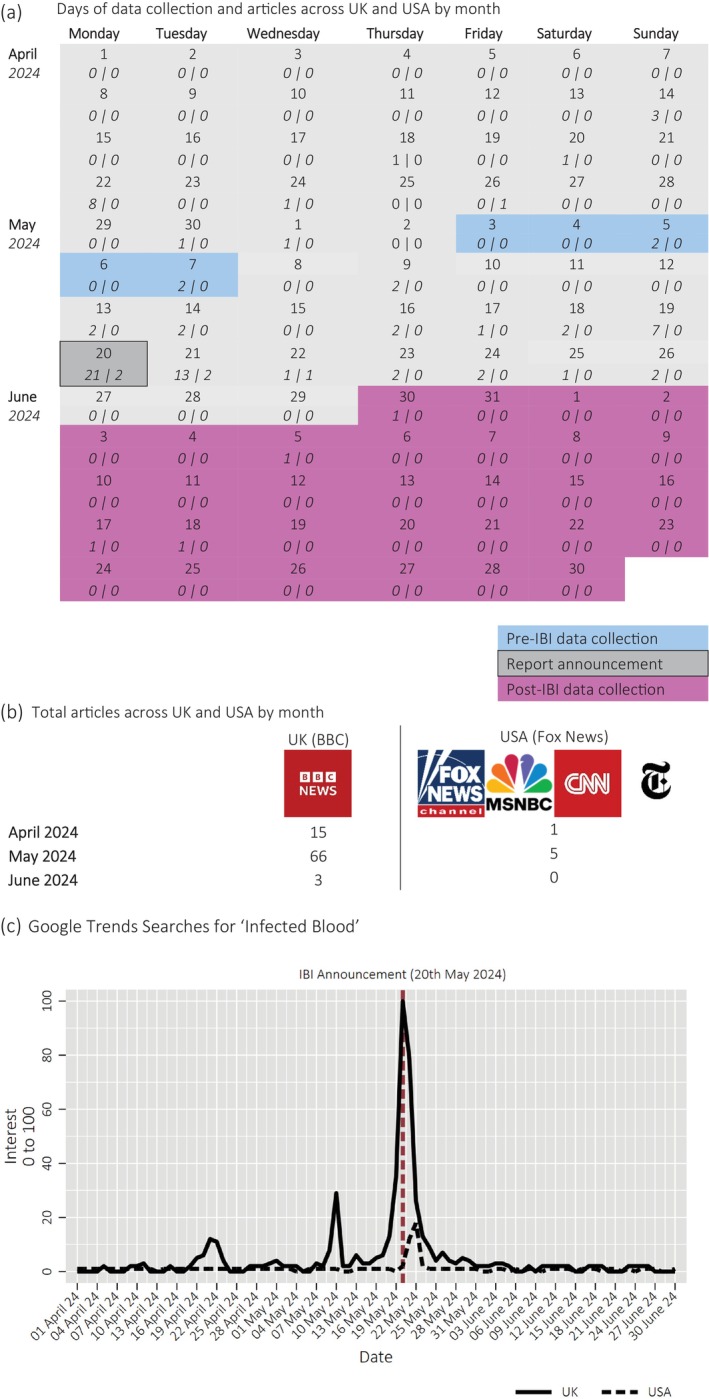
Data Collection Timelines and Media Coverage Across UK and USA. (A) ‘Pre‐IBI’ data collection (03/05–07/05), ‘Post‐IBI’ data collection (30/05–30/06). Post‐IBI data collection was longer as participants needed additional time to complete the follow‐up survey. (B) The number of articles were observed through the following search terms on Google (www.google.com) between 01/04 and 30/06: ‘site:www.bbc.com “infected blood”’, ‘site:www.msnbc.com “infected blood”’, ‘site:www.foxnews.com “infected blood”’, and ‘site:www.cnn.com “infected blood”’. (C) Google Trends search data for the UK/USA for the term ‘Infected Blood’. The *y‐axis* refers to Google's ‘Interest’ index, described as: ‘Numbers represent search interest relative to the highest point on the chart for the given region and time. A value of 100 is the peak popularity for the term. A value of 50 means that the term is half as popular. A score of 0 means there was not enough data for this term’. The graph shows maximal interest in the UK around the IBI announcement (20th May 2024), with a sharp drop‐off afterwards. There is relatively low interest across the USA, with a small spike a few days after the IBI announcement. The data to produce these graphs can be accessed from the following Google Trends link.

Figure 3 (Panel A) shows the timelines of data collection and (Panel B) media coverage across the UK (BBC) and the USA (Fox News, MSNBC, CNN and NYT). Media surrounding the IBI was substantial in the UK, and minimal in the USA. In the UK, there was moderate activity in the months leading up to the announcement, with 15 articles published in April 2024, rising to 66 in May and a substantial drop‐off in June with only three articles. Around the time of the IBI announcement, the nature of these articles varied addressing (i) *the cover‐up* (‘Infected blood inquiry finds scandal “was not an accident”’); (ii) *Personal impact stories* of both victims and families (‘Infected blood: “They put my whole family at risk”)’; (iii) *Compensation and justice* (‘Infected blood scandal: Sunak promises “comprehensive” blood compensation’); (iv) *Government and political reactions* (‘Rishi Sunak: “Unequivocal apology” for victims of infected blood scandal’); and (v) *Regional and institutional failures* (“Welsh minister apology for infected blood 'tragedy‘). Of the 84 articles in April, May, and June 2024, 60 were written between the two data collection points (08/05–29/05), representing a significant proportion of the total media surrounding the topic (>70%).

Data from Google Trends surrounding the search term for ‘Infected Blood’ (Figure 3, Panel C) shows that the UK saw significantly higher relative rates of search activity than the USA, particularly around the time of the IBI announcement (20th May 2024). In fact, there was more than five times the relative interest for the term in the UK (Index = 100, 20th May 2024) than in the USA (Index = 18, 22nd May 2024). This data further supports the argument that the USA saw relatively little media coverage on the issue.

## 4 DISCUSSION

Despite the significant implications of the IBI findings, the UK participants in our sample continue to see the safety of its blood supply as very high. In fact, UK blood is perceived as safer by UK participants than American blood is perceived by US participants. Following the IBI announcement, during June, there was a significant but very small (1.83%) decline in the perceived safety of the UK blood supply by people in the UK, but no change in perceived transfusion infection risk. Thus, while there was a statistically significant decline in perceived safety, overall, the UK blood supply remains seen as very safe. While more people in the UK (34.6%) than in the US (28.4%) showed a decrease in perceived safety when we consider the number of people who showed a significant decline in perceived safety, it is small: 3.3% in the UK and 4.4% in the USA.

This relatively small impact is likely a result of several factors. First, it indicates that the public may feel that the inquiry was comprehensive, open and fair. Indeed, Sir Brian Langstaff, instrumental in putting the report together, received a standing ovation after announcing its release.^49^ Moreover, the speech in the House of Commons reaffirmed the government's commitment to enacting legislation to compensate those affected by the scandal with mutual support across the parties.^50^, ^51^ Second, it is clear that UK blood services have changed their practices extensively since the 1970–90s, and blood in the UK today is very safe.^1^, ^52^, ^53^ Third, it is also possible that this minimal impact in the general population sample is a function of the large drop‐off in media coverage observed in June 2024, which may have been a result of other major political events pulling focus away from the IBI, such as the announcement of a UK general election (22nd May 2024), and the continued developments of the Post Office Horizon scandal.^54^

In terms of behavioural impact, we observe that those showing a significant decline in perceived safety are less willing to donate blood or encourage others to donate in the full sample and the USA. However, in the UK, we only observe a significant effect in encouraging others. Thus, it may be that in the UK, while people may feel that they would not be put off donating, they would not want to take responsibility for encouraging others to donate. These results are consistent with previous research, showing the positive relationship between the perception of safety and willingness to donate.^25^, ^55^ However, the relatively weaker response in the UK suggests that while perceptions of the safety of the blood supply might have been slightly shaken, they do not drastically alter the general public's overall willingness to donate blood, potentially due to the aforementioned factors.

While there are clear overall effects, there are also important demographic variations. Women are more likely to feel that the infection risk has risen post‐IBI reporting, older people feel that safety has reduced, and non‐donors are more likely to be put off donating and asking others to donate.

These findings clearly demonstrate that the IBI announcement had minimal impact on the UK general public, aside from a slight decrease in perceived safety. Consequently, NHSBT can be reassured that the overall psychological and behavioural effects of the IBI inquiry on the general population are minimal. However, it is important to acknowledge that blood recipients, particularly those requiring multiple transfusions over their lifetime, have experienced and continue to experience significant impacts. The work reported here is not intended to undermine or diminish the concerns and experiences of this specific group. Indeed, the work reported here focused solely on the general public who had not received a transfusion. Future research needs to be undertaken to explore the impact of the IBI on blood recipients, especially those requiring multiple transfusions.

### 4.1 Limitations and future directions

There may be some selection bias (i.e., attrition), but this is unlikely given that the follow‐up response rates are all about 85%.^27^ However, it must be noted that the US sample is slightly underpowered; and we also acknowledge that the study only evaluates the ‘short‐term’ impacts of the IBI announcement and that longer‐term evaluation and studies exploring the impact on current blood recipients and victims are needed. More objective data from NHSBT surrounding the number of registrations and successful donations would better determine the direct impacts on the organisation. Finally, while we examined perceptions of overall blood safety for transfusion, future work should consider differentiating between blood, blood products and plasma.

This work provides an initial evaluation surrounding the impact of the IBI. We aim to follow up with participants from this sample over the coming years to explore longer‐term impacts. We also plan to use the same questions to explore the impact of the IBI on recipient populations, particularly as the implications of the IBI are realised over the coming years (i.e., as compensation is provided to victims and their families).

## 5 CONCLUSIONS

The general public in the UK perceives the current blood supply as extremely safe, and the IBI announcement had a minimal impact on this perception. Compared to the UK, blood safety perceptions are lower in the USA. Moreover, significant reductions in safety perceptions are associated with a lower willingness to donate blood. However, in the UK, even those who perceive a reduction in safety do not show a significantly lower willingness to donate. Future research should explore long‐term impacts, continue to monitor public perceptions as compensation schemes are rolled out, and examine opinions and perceptions of blood recipients.

## AUTHOR CONTRIBUTIONS

Richard Mills, Eamonn Ferguson, Barbara Masser, and Eva‐Maria Merz set up the survey, and Richard Mills and Eamonn Ferguson analysed the data and wrote the manuscript. Robert Smith researched the media impact surrounding the IBI. Eva‐Maria Merz, Mark Croucher, Barbara Masser, Robert Smith and Susan R. Brailsford provided feedback on the analysis and comments on several iterations of the draft.

## FUNDING INFORMATION

This work was funded by the National Institute of Health and Care Research (NIHR) (grant number NIHR203337).

## CONFLICT OF INTEREST STATEMENT

The authors have no competing interests.

## ETHICS STATEMENT

This research was reviewed and approved by the Ethics Review Board in the School of Psychology, University of Nottingham (F1351: 30/05/2022; F1366: 06/07/2022; F1411: 03/02/2023; F1422: 22/02/2023).

## PARTICIPANT CONSENT STATEMENT

All participants were over 18 years old and provided full informed consent.

## Supporting information


Data S1.

## Data Availability

Data and analysis files will be made freely available on the Open Science Framework.
