# Supplementary material for: The infected blood inquiry: Impact on public perceptions of blood supply risk, safety, and donation attitudes
Source: Transfus Med. 2024 Nov 12;34(6):478–90. doi: 10.1111/tme.13108 (PMC11653061; doi:10.1111/tme.13108)
Supplement: Supplementary file 1 — Data S1. [file TME-34-478-s001.docx]

**Supplementary File for “The Infected Blood Inquiry: Impact on Public Perceptions of Blood Supply Risk, Safety, and Donation Attitudes”**

Contents

[Prolific survey questions 2](#_Toc178786127)

[Relevant Pre-IBI survey (UK) 2](#_Toc178786128)

[Relevant Pre-IBI survey (USA) 5](#_Toc178786129)

[Relevant Post-IBI survey debrief 8](#_Toc178786130)

[Results 10](#_Toc178786131)

[Summary statistics 10](#_Toc178786132)

[Pairwise correlations 13](#_Toc178786133)

[Main regressions: 16](#_Toc178786134)

[Simple change index: 18](#_Toc178786135)

[Restricted sample: 20](#_Toc178786136)

# Prolific survey questions

The pre-IBI questionnaire questions were part of a larger set of questions on perceptions surrounding incentives in the USA and UK. This study is preregistered on Open Science Framework (for more details, see: [OSF link](https://osf.io/6wmqe/?view_only=6079b641bdbe4c9699d4565337c331bf)). Among others, the pre-IBI questionnaire also asks for a comprehensive set of demographic information, which is not asked in the post-IBI survey (to ensure the follow-up study was not too long).

Note that given the contexts, the USA and UK had their differences in respective spelling and slightly different demographic questions.

## Relevant Pre-IBI survey (UK)

#### Demographic questions:

*1. How old are you in years?*

*2. What is your sex? (a question on your gender identity will follow)*

*• Female*

*• Male*

*• Prefer not to say*

*Display This Question:*

*If Q2 = No*

*3. Please provide your gender identity*

*• Trans woman*

*• Trans man*

*• Non-binary*

*• Other gender identity (if selected, please enter preference)*

*• Prefer not to say*

*4. What ethnic group best describes you?*

*• Asian or Asian British (3)*

*• Black, African, Caribbean or Black British (4)*

*• Mixed or multiple ethnic groups (2)*

*• White (1)*

*• Other (5) ______________*

*• I prefer not to say (-9)*

*Display This Question:*

*If ethnicity = White*

*Which of the following best describes your White background?*

*• English, Welsh, Scottish, Northern Irish or British (1)*

*• Irish (2)*

*• Gypsy or Irish Traveller (3)*

*• Any other White background (optional) (4) ______________*

*• Other (-9)*

*Display This Question:*

*If ethnicity = Mixed or multiple ethnic groups*

*Which of the following best describes your mixed or multiple ethnic groups background?*

*• White and Black Caribbean (1)*

*• White and Black African (2)*

*• White and Asian (3)*

*• Any other mixed or multiple ethnic background (optional) (4) ______________*

*• Other (-9)*

*Display This Question:*

*If ethnicity = Asian or Asian British*

*Which of the following best describes your Asian or Asian British background?*

*• Indian (1)*

*• Pakistani (2)*

*• Bangladeshi (3)*

*• Chinese (4)*

*• Any other Asian background (5) ______________*

*• Other (-9)*

*5. What is your household pre-tax income last year?*

*• Less than £20,000 (1)*

*• £20,000-£39,999 (2)*

*• £40,000-£59,999 (3)*

*• £60,000-£99,999 (4)*

*• £100,000-£149,999 (4)*

*• More than £150,000 (5)*

*• Prefer not to say (6)*

*6. Including yourself, how many people currently live in your household? [numeric entry]*

*7. What is the highest level of education you have achieved?*

*• Left school with no qualifications*

*• GCSEs, O-Levels, or equivalent*

*• A-Levels, BTEC, Scottish Highers, or equivalent*

*• Foundation degree, Higher National Diploma (HND), or equivalent vocational qualifications*

*• Undergraduate/Bachelors degree*

*• Postgraduate degree (e.g., Master’s, PhD, Professional qualifications like LLB, LPC, MBBS, BDS, MBA)*

*• Prefer not to say*

*8. Here is a 7-point scale on which the political views that people might hold are arranged from extremely liberal (left) to extremely conservative (right). Where would you place yourself on this scale?*

*Political ideology [Extremely liberal - 0, 1, 2, 3, 4, 5, 6, 7 – Extremely Conservative]*

*9. In which country do you currently live in?*

*▼ Afghanistan (1) ... Zimbabwe (1357)*

*10. In which region do you currently live in the UK?*

*▼ Yorkshire and The Humber (4) ... I do not reside in the United Kingdom (16)*

#### Donation history:

*1. Have you ever been a recipient of blood or blood products?*

*• Yes*

*• No*

*• I’m not sure*

*• Prefer not to say*

*2. Have you ever donated blood?*

*• Yes*

*• No*

*• I’m not sure*

*• Prefer not to say*

*3. When did you last donate blood?*

*• Within the last month*

*• 1 to 6 months ago*

*• 6 months to 1 year ago*

*• 1 to 2 years ago*

*• Longer than 2 years ago*

*• I cannot remember*

*• Prefer not to say*

*4. Roughly how many times have you donated blood?*

*[Numeric entry]*

*5. Have you ever donated plasma?*

*• Yes*

*• No*

*• I’m not sure*

*• Prefer not to say*

*6. Roughly how many times have you donated plasma?*

*[Numeric entry]*

#### Risk & Safety:

*1. What do you feel the level of infection risk is to a patient receiving blood in the UK?*

*[no risk at all - 1, 2, 3, 4, 5 - an extremely large risk]*

*2. What is the acceptable level of infection risk to a patient receiving blood in the UK?*

*[no risk at all - 1, 2, 3, 4, 5 - an extremely large risk]*

*3. To what extent do you feel it is safe in the UK to have a blood transfusion if you need one?*

*[not at all safe - 1, 2, 3, 4, 5, 6, 7, 8 ,9 10, 11 – completely safe]*

#### Willingness to donate and encourage others to donate:

Please read the following statements and indicate the degree to which the statement applies to you from 1 = "Not at all" to 7 = "Completely."

1. I am willing to donate blood (assuming you are eligible)

2. I am willing to encourage others to donate blood

## Relevant Pre-IBI survey (USA)

#### Demographic questions:

*1. How old are you in years?*

*2. What is your sex? (a question on your gender identity will follow)*

*• Female*

*• Male*

*• Prefer not to say*

*Display This Question:*

*If Q2 = No*

*3. Please provide your gender identity*

*• Trans woman*

*• Trans man*

*• Non-binary*

*• Other gender identity (if selected, please enter preference)*

*• Prefer not to say*

*4a. Please select all categories that to describe your racial background:*

*• American Indian or Alaska Native*

*• Black or African American*

*• Asian*

*• Native Hawaiian or Other Pacific Islander*

*• White or European American or Middle Eastern American*

*• I prefer a different descriptor (if selected, please enter preference)*

*• I prefer not to say*

*4b. Please choose a category that best describes your ethnicity:*

*• Hispanic or Latino*

*• Not Hispanic or Latino*

*• I prefer a different descriptor (if selected, please enter preference)*

*• I prefer not to say*

*5. What is your household pre-tax income last year?*

*• Less than $25,000 (1)*

*• $25,000-$49,999 (2)*

*• $50,000-$74,999 (3)*

*• $75,000-$124,999 (4)*

*• $125,000-$187,499 (4)*

*• More than $187,500 (5)*

*• Prefer not to say (6)*

*6. Including yourself, how many people currently live in your household? [numeric entry]*

*[Converted £ to $ at a 1.25 exchange rate]*

*7. What is the highest level of education you have achieved?*

*• Left school with no qualifications*

*• High school diploma or GED*

*• Some college, but no degree*

*• Associate's degree or technical degree*

*• Bachelor's degree*

*• Graduate or professional degree (e.g., MA, MS, MBA, PhD, JD, MD, DDS, etc.)*

*• Prefer not to say*

*8. Here is a 7-point scale on which the political views that people might hold are arranged from extremely liberal (left) to extremely conservative (right). Where would you place yourself on this scale?*

*Political ideology [Extremely liberal - 0, 1, 2, 3, 4, 5, 6, 7 – Extremely Conservative]*

*9. In which country do you currently live in?*

*▼ Afghanistan (1) ... Zimbabwe (1357)*

*10. In which state do you currently live in the USA?*

*▼ Alabama (1) ... I do not reside in the United States (53)*

#### Donation history:

*1. Have you ever been a recipient of blood or blood products?*

*• Yes*

*• No*

*• I’m not sure*

*• Prefer not to say*

*2. Have you ever donated blood?*

*• Yes*

*• No*

*• I’m not sure*

*• Prefer not to say*

*3. When did you last donate blood?*

*• Within the last month*

*• 1 to 6 months ago*

*• 6 months to 1 year ago*

*• 1 to 2 years ago*

*• Longer than 2 years ago*

*• I cannot remember*

*• Prefer not to say*

*4. Roughly how many times have you donated blood?*

*[Numeric entry]*

*5. Have you ever donated plasma?*

*• Yes*

*• No*

*• I’m not sure*

*• Prefer not to say*

*6. Roughly how many times have you donated plasma?*

*[Numeric entry]*

#### Risk & Safety:

*1. What do you feel the level of infection risk is to a patient receiving blood in the USA?*

*[no risk at all - 1, 2, 3, 4, 5 - an extremely large risk]*

*2. What is the acceptable level of infection risk to a patient receiving blood in the USA?*

*[no risk at all - 1, 2, 3, 4, 5 - an extremely large risk]*

*3. To what extent do you feel it is safe in the USA to have a blood transfusion if you need one?*

*[not at all safe - 1, 2, 3, 4, 5, 6, 7, 8 ,9 10, 11 – completely safe]*

#### Willingness to donate and encourage others to donate:

Please read the following statements and indicate the degree to which the statement applies to you from 1 = "Not at all" to 7 = "Completely."

1. I am willing to donate blood (assuming you are eligible)

2. I am willing to encourage others to donate blood

## Relevant Post-IBI survey debrief

Since the post-IBI survey questions were identical, except for demographics not being asked, we do not repeat them here. However, importantly, we do describe the debrief statement after all participants completed the follow-up survey as we wanted to ensure subjects were as informed as possible about the nature of the study.

#### Debrief (UK)

**Title:** Short follow-up to “Categorisation of incentives to encourage blood donation”

**Research fellow:** Richard Mills: [richard.mills2@nottingham.ac.uk](mailto:richard.mills2@nottingham.ac.uk)

**Principal investigator:** Eamonn Ferguson: [eamonn.ferguson@nottingham.ac.uk](mailto:eamonn.ferguson@nottingham.ac.uk)

**Ethics reference:** F1523, F1534 and F1540

**Rationale:** This short-follow study is designed to understand how people perceive the safety of blood, trust and willingness to donate blood, particularly in relation to the recent announcement surrounding the outcome of the Infected Blood Inquiry (IBI) in the UK.

**Background Information:** The IBI is an independent public statutory investigation focused on the circumstances under which men, women, and children who received treatment from UK national health services were administered infected blood and blood products, particularly during the 1970s and 1980s. The final report was published on 20th May 2024.

For those interested in finding out more information, please visit: [IBI Information](https://www.nhsbt.nhs.uk/who-we-are/transparency/infected-blood-inquiry/)

For frequently asked questions, please see: [IBI FAQs](https://www.nhsbt.nhs.uk/who-we-are/transparency/infected-blood-inquiry/frequently-asked-questions/)

It should be noted that modern safety standards have improved enormously, and today, blood in the UK is among the safest in the world:

[WHO blood safety](https://www.who.int/news-room/fact-sheets/detail/blood-safety-and-availability)

[NHSBT blood safety](https://www.blood.co.uk/the-donation-process/further-information/your-safety/)

Data Analysis: Responses will be analysed using standard statistical procedures to understand better how incentives to encourage blood donation are perceived.

If you have any concerns or complaints about the study, please contact Stephen Jackson (Ethics Committee Chair): [stephen.jackson@nottingham.ac.uk](mailto:stephen.jackson@nottingham.ac.uk)

Please click on the arrow below to complete and submit the survey and thank you for your participation.

#### Debrief (USA)

**Title:** Short follow-up to “Categorization of incentives to encourage blood donation”

**Research fellow:** Richard Mills: [richard.mills2@nottingham.ac.uk](mailto:richard.mills2@nottingham.ac.uk)

**Principal investigator:** Eamonn Ferguson: [eamonn.ferguson@nottingham.ac.uk](mailto:eamonn.ferguson@nottingham.ac.uk)

**Ethics reference:** F1523, F1534 and F1540

**Rationale:** This short-follow study is designed to understand how people perceive the safety of blood, trust and willingness to donate blood, particularly in relation to the recent announcement surrounding the outcome of the Infected Blood Inquiry (IBI) in the UK.

**Background Information:** The IBI is an independent public statutory investigation focused on the circumstances under which men, women, and children who received treatment from UK national health services were administered infected blood and blood products, particularly during the 1970s and 1980s. The final report was published on 20th May 2024.

For those interested in finding out more information, please visit: [IBI Information](https://www.nhsbt.nhs.uk/who-we-are/transparency/infected-blood-inquiry/)

For frequently asked questions, please see: [IBI FAQs](https://www.nhsbt.nhs.uk/who-we-are/transparency/infected-blood-inquiry/frequently-asked-questions/)

It should be noted that modern safety standards have improved enormously, and today, blood in the UK and the USA is among the safest in the world:

[WHO blood safety](https://www.who.int/news-room/fact-sheets/detail/blood-safety-and-availability)

[AABB blood safety](https://www.aabb.org/for-donors-patients/about-blood-donation/how-the-blood-community-ensures-the-safety-of-blood-in-the-united-states)

**Background Information:** The IBI is an independent public statutory investigation focused on the circumstances under which men, women, and children who received treatment from UK national health services were administered infected blood and blood products, particularly during the 1970s and 1980s. The final report was published on 20th May 2024.

Data Analysis: Responses will be analyzed using standard statistical procedures to understand better how incentives to encourage blood donation are perceived.

If you have any concerns or complaints about the study, please contact Stephen Jackson (Ethics Committee Chair): [stephen.jackson@nottingham.ac.uk](mailto:stephen.jackson@nottingham.ac.uk)

Please click on the arrow below to complete and submit the survey and thank you for your participation.

# Results

## Summary statistics

***Table S1*: Sample Summary statistics**

|  | Pre-IBI | Post-IBI | Total |
| --- | --- | --- | --- |
| N(%) | 1,635 (50.0%) | 1,635 (50.0%) | 3,270 (100.0%) |
| Country |  |  |  |
| USA | 747 (45.7%) | 747 (45.7%) | 1,494 (45.7%) |
| UK | 888 (54.3%) | 888 (54.3%) | 1,776 (54.3%) |
| Note: A total of 175 observations were not collected (this excludes recipients of blood, which excludes 282 participants who stated they had received blood or blood products before). These statistics show that relative to pre-IBI, 95% of the UK sample responded to the post-IBI follow-up survey. For the USA, there was an 85% response rate to the follow-up. | | | |

***Table S2*: Sample Summary statistics for Attrition**

|  | Did not complete Post-IBI survey | Completed Post-IBI survey | Total | p-value |
| --- | --- | --- | --- | --- |
| N(%) | 148 (4.2%) | 3,414 (95.8%) | 3,562 (100.0%) |  |
| Age | 37.544 (13.203) | 42.863 (13.359) | 42.644 (13.393) | **<0.001***** |
| Female | 0.459 (0.500) | 0.511 (0.500) | 0.509 (0.500) | 0.219 |
| Education |  |  |  |  |
| No qualifications | 0 (0.0%) | 30 (0.9%) | 30 (0.8%) | 0.125 |
| High school | 12 (8.2%) | 436 (12.8%) | 448 (12.6%) |  |
| Some college | 28 (19.0%) | 640 (18.8%) | 668 (18.8%) |  |
| Associate Degree | 7 (4.8%) | 303 (8.9%) | 310 (8.7%) |  |
| Bachelors Degree | 70 (47.6%) | 1,378 (40.4%) | 1,448 (40.7%) |  |
| Postgraduate Degree | 30 (20.4%) | 620 (18.2%) | 650 (18.3%) |  |
| Blood donor history |  |  |  |  |
| Non-donor | 72 (49.0%) | 1,740 (51.1%) | 1,812 (51.0%) | 0.609 |
| Donor | 75 (51.0%) | 1,663 (48.9%) | 1,738 (49.0%) |  |
| **Notes:** p-value from two-sample t-test (for continuous variables) or Chi-squared tests (for proportions). The table shows that younger people are more likely to drop-out of the sample. | | | | |

**Table *S3*:** **Summary statistics of main variables**

1. Summary statistics (UK)

|  | Pre-IBI | Post-IBI | Total | p-value |
| --- | --- | --- | --- | --- |
| N(%) | 888 (50.0%) | 888 (50.0%) | 1,776 (100.0%) |  |
| Infection Risk Patient | 2.232 (0.805) | 2.234 (0.776) | 2.233 (0.790) | 0.957 |
| Transfusion Safety | 8.734 (1.783) | 8.569 (1.815) | 8.652 (1.801) | **0.006***** |
|  |  |  |  |  |
| Approach | 5.396 (1.739) | 5.375 (1.736) | 5.386 (1.737) | 0.585 |
| Encourage | 5.264 (1.646) | 5.161 (1.695) | - 1. .671) | **0.018**** |

**Notes:** Significance is determined by several ordinary least squares regressions (controlling for additional characteristics such as age, gender, education, political status, prior donor status and region/states residing in), clustered at the individual level to account for repeated observations over time.

1. Summary statistics (USA)

|  | Pre-IBI | Post-IBI | Total | p-value |
| --- | --- | --- | --- | --- |
| N(%) | 747 (50.0%) | 747 (50.0%) | 1,494 (100.0%) |  |
| Infection Risk Patient | 2.301 (0.869) | 2.224 (0.849) | 2.263 (0.860) | **0.005***** |
| Transfusion Safety | 8.239 (2.125) | 8.278 (2.107) | 8.258 (2.115) | 0.620 |
|  |  |  |  |  |
| Approach | 5.481 (1.660) | 5.459 (1.703) | 5.470 (1.681) | 0.525 |
| Encourage | 5.357 (1.687) | 5.327 (1.728) | 5.343 (1.707) | **0.527** |

**Notes:** Significance is determined by several ordinary least squares regressions (controlling for additional characteristics such as age, gender, education, political status, prior donor status and region/states residing in), clustered at the individual level to account for repeated observations over time.

**Figure S1: Histogram of post-IBI data collection dates**


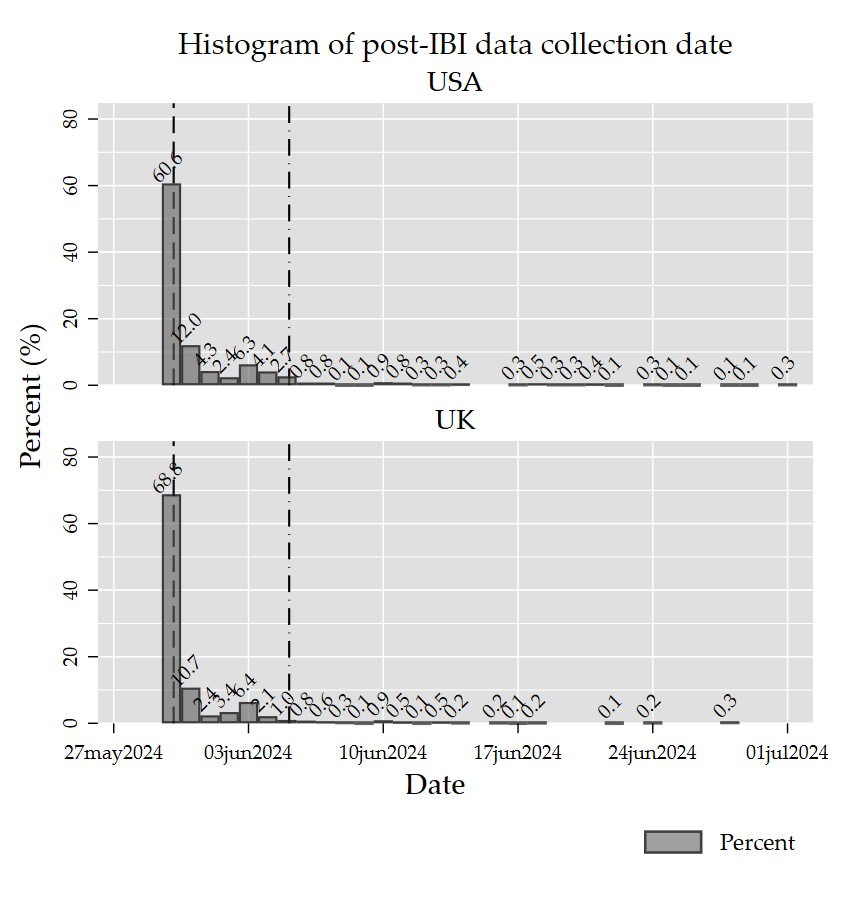


**Notes:** Post-IBI data collection took place between 30^th^ May and 31^st^ June 2024. This period was substantially longer than the Pre-IBI data collection between 2^nd^ and 7^th^ May 2024, due to following up the same individuals. Though, as evident in these histograms, the majority of data (97.41% for UK and 96.25% for USA) in the first week of data collection (i.e, 30^th^ May to 5^th^ June 2024).

## Pairwise correlations

**Table *S4*:** **Pairwise correlations of main variables (UK)**

| Variables | Infection Risk (Pre-IBI) | Infection Risk (Post-IBI) | Safety (Pre-IBI) | Safety (Post-IBI) | Approach (Pre-IBI) | Approach (Post-IBI) | Encourage (Pre-IBI) | Encourage (Post-IBI) |
| --- | --- | --- | --- | --- | --- | --- | --- | --- |
| Infection Risk (Pre-IBI) | 1.000 |  |  |  |  |  |  |  |
|  |  |  |  |  |  |  |  |  |
| Infection Risk (Post-IBI) | 0.517*** | 1.000 |  |  |  |  |  |  |
|  | (0.000) |  |  |  |  |  |  |  |
| Safety (Pre-IBI) | -0.538*** | -0.437*** | 1.000 |  |  |  |  |  |
|  | (0.000) | (0.000) |  |  |  |  |  |  |
| Safety (Post-IBI) | -0.429*** | -0.525*** | 0.514*** | 1.000 |  |  |  |  |
|  | (0.000) | (0.000) | (0.000) |  |  |  |  |  |
| Approach (Pre-IBI) | -0.151*** | -0.125*** | 0.239*** | 0.207*** | 1.000 |  |  |  |
|  | (0.000) | (0.000) | (0.000) | (0.000) |  |  |  |  |
| Approach (Post-IBI) | -0.137*** | -0.132*** | 0.235*** | 0.249*** | 0.785*** | 1.000 |  |  |
|  | (0.000) | (0.000) | (0.000) | (0.000) | (0.000) |  |  |  |
| Encourage (Pre-IBI) | -0.165*** | -0.165*** | 0.328*** | 0.265*** | 0.624*** | 0.536*** | 1.000 |  |
|  | (0.000) | (0.000) | (0.000) | (0.000) | (0.000) | (0.000) |  |  |
| Encourage (Post-IBI) | -0.160*** | -0.148*** | 0.236*** | 0.302*** | 0.546*** | 0.628*** | 0.714*** | 1.000 |
|  | (0.000) | (0.000) | (0.000) | (0.000) | (0.000) | (0.000) | (0.000) |  |
| **Notes:** **** p<0.01, ** p<0.05, * p<0.1* | | | | | | | | |

**Table *S5*: Pairwise correlations of main variables (USA)**

| Variables | Infection Risk (Pre-IBI) | Infection Risk (Post-IBI) | Safety (Pre-IBI) | Safety (Post-IBI) | Approach (Pre-IBI) | Approach (Post-IBI) | Encourage (Pre-IBI) | Encourage (Post-IBI) |
| --- | --- | --- | --- | --- | --- | --- | --- | --- |
| Infection Risk (Pre-IBI) | 1.000 |  |  |  |  |  |  |  |
|  |  |  |  |  |  |  |  |  |
| Infection Risk (Post-IBI) | 0.517*** | 1.000 |  |  |  |  |  |  |
|  | (0.000) |  |  |  |  |  |  |  |
| Safety (Pre-IBI) | -0.538*** | -0.437*** | 1.000 |  |  |  |  |  |
|  | (0.000) | (0.000) |  |  |  |  |  |  |
| Safety (Post-IBI) | -0.429*** | -0.525*** | 0.514*** | 1.000 |  |  |  |  |
|  | (0.000) | (0.000) | (0.000) |  |  |  |  |  |
| Approach (Pre-IBI) | -0.151*** | -0.125*** | 0.239*** | 0.207*** | 1.000 |  |  |  |
|  | (0.000) | (0.000) | (0.000) | (0.000) |  |  |  |  |
| Approach (Post-IBI) | -0.137*** | -0.132*** | 0.235*** | 0.249*** | 0.785*** | 1.000 |  |  |
|  | (0.000) | (0.000) | (0.000) | (0.000) | (0.000) |  |  |  |
| Encourage (Pre-IBI) | -0.165*** | -0.165*** | 0.328*** | 0.265*** | 0.624*** | 0.536*** | 1.000 |  |
|  | (0.000) | (0.000) | (0.000) | (0.000) | (0.000) | (0.000) |  |  |
| Encourage (Post-IBI) | -0.160*** | -0.148*** | 0.236*** | 0.302*** | 0.546*** | 0.628*** | 0.714*** | 1.000 |
|  | (0.000) | (0.000) | (0.000) | (0.000) | (0.000) | (0.000) | (0.000) |  |
| **Notes:** **** p<0.01, ** p<0.05, * p<0.1* | | | | | | | | |

**Table *S6*: Pairwise correlations of main variables (Total)**

| Variables | Infection Risk (Pre-IBI) | Infection Risk (Post-IBI) | Safety (Pre-IBI) | Safety (Post-IBI) | Approach (Pre-IBI) | Approach (Post-IBI) | Encourage (Pre-IBI) | Encourage (Post-IBI) |
| --- | --- | --- | --- | --- | --- | --- | --- | --- |
| Infection Risk (Pre-IBI) | 1.000 |  |  |  |  |  |  |  |
|  |  |  |  |  |  |  |  |  |
| Infection Risk (Post-IBI) | 0.539*** | 1.000 |  |  |  |  |  |  |
|  | (0.000) |  |  |  |  |  |  |  |
| Safety (Pre-IBI) | **-0.505***** | -0.442*** | 1.000 |  |  |  |  |  |
|  | (0.000) | (0.000) |  |  |  |  |  |  |
| Safety (Post-IBI) | -0.430*** | **-0.518***** | 0.564*** | 1.000 |  |  |  |  |
|  | (0.000) | (0.000) | (0.000) |  |  |  |  |  |
| Approach (Pre-IBI) | -0.132*** | -0.124*** | 0.230*** | 0.184*** | 1.000 |  |  |  |
|  | (0.000) | (0.000) | (0.000) | (0.000) |  |  |  |  |
| Approach (Post-IBI) | -0.108*** | -0.133*** | 0.217*** | 0.232*** | 0.773*** | 1.000 |  |  |
|  | (0.000) | (0.000) | (0.000) | (0.000) | (0.000) |  |  |  |
| Encourage (Pre-IBI) | -0.125*** | -0.126*** | 0.246*** | 0.200*** | 0.621*** | 0.518*** | 1.000 |  |
|  | (0.000) | (0.000) | (0.000) | (0.000) | (0.000) | (0.000) |  |  |
| Encourage (Post-IBI) | -0.107*** | -0.119*** | 0.192*** | 0.236*** | 0.525*** | 0.627*** | 0.702*** | 1.000 |
|  | (0.000) | (0.000) | (0.000) | (0.000) | (0.000) | (0.000) | (0.000) |  |
| **Notes:** **** p<0.01, ** p<0.05, * p<0.1* | | | | | | | | |

## Main regressions:

**Table S7: Ordinary Least Squares by Country (Infection Risk)**

|  | USA | UK |
| --- | --- | --- |
| VARIABLES | OLS II | OLS IV |
|  |  |  |
| Post-IBI | **-0.103***** | 0.002 |
|  | (0.037) | (0.032) |
|  |  |  |
| Constant | 1.050** | 0.191 |
|  | (0.531) | (0.285) |
|  |  |  |
| Region/State controls | ✓ | ✓ |
| Observations | 1,473 | 1,754 |
| R-squared | 0.072 | 0.032 |
| Number of Observations | 1473 | 1754 |
| Dof | 52 | 20 |

**Notes:** Ordinary Least Squares (OLS) regressions, with infection risk as the dependent variables across USA/UK. Post-IBI takes on a value of 0, 1 (0 = Pre-IBI, 1= Post-IBI). Additional controls include age, gender, education, prior donor status and region/states residing in), clustered at the individual level to account for repeated observations over time. Cluster robust standard errors in parenthesis *** p < 0.01; ** p < 0.05; * p < 0.1

**Table S8: Ordinary Least Squares Country Interaction (Infection Risk)**

|  | (1) | (2) | (3) | (4) |
| --- | --- | --- | --- | --- |
| VARIABLES | OLS I | OLS II | OLS III | OLS IV |
|  |  |  |  |  |
| UK | -0.036 | -0.084* | -0.056 | **-0.108**** |
|  | (0.044) | (0.051) | (0.045) | (0.052) |
| Post-IBI | -0.041* | **-0.093***** | -0.046* | **-0.102***** |
|  | (0.024) | (0.036) | (0.024) | (0.036) |
| UK x Post-IBI |  | **0.096**** |  | **0.104**** |
|  |  | (0.048) |  | (0.048) |
|  |  |  |  |  |
| Constant | 0.040 | 0.066* | 0.609** | 0.637** |
|  | (0.036) | (0.039) | (0.265) | (0.265) |
|  |  |  |  |  |
| Observations | 3,261 | 3,261 | 3,237 | 3,237 |
| R-squared | 0.001 | 0.001 | 0.014 | 0.015 |
| Number of Observations | 3261 | 3261 | 3237 | 3237 |
| Dof | 2 | 3 | 10 | 11 |

**Notes:** Ordinary Least Squares (OLS) regressions, with infection risk as the dependent variables across. UK takes on a value of 0,1 (0=USA, 1=UK). Post-IBI takes on a value of 0, 1 (0 = Pre-IBI, 1= Post-IBI). Additional controls include age, gender, education, and prior donor status), clustered at the individual level to account for repeated observations over time. Cluster robust standard errors in parenthesis *** p < 0.01; ** p < 0.05; * p < 0.1

**Table S9: Ordinary Least Squares by Country (Safety)**

|  | USA | UK |
| --- | --- | --- |
| VARIABLES | OLS II | OLS IV |
|  |  |  |
| Post-IBI | 0.018 | **-0.086***** |
|  | (0.036) | (0.031) |
|  |  |  |
| Constant | -0.934 | -0.116 |
|  | (0.671) | (0.348) |
|  |  |  |
| Region/State controls | ✓ | ✓ |
| Observations | 1,472 | 1,755 |
| R-squared | 0.059 | 0.035 |
| Number of Observations | 1472 | 1755 |
| Dof | 52 | 20 |

**Notes:** Ordinary Least Squares (OLS) regressions, with safety as the dependent variables across USA/UK. Post-IBI takes on a value of 0, 1 (0 = Pre-IBI, 1= Post-IBI). Additional controls include age, gender, education, prior donor status and region/states residing in), clustered at the individual level to account for repeated observations over time. Cluster robust standard errors in parenthesis *** p < 0.01; ** p < 0.05; * p < 0.1

**Table S10: Ordinary Least Squares Country Interaction (Safety)**

|  | (1) | (2) | (3) | (4) |
| --- | --- | --- | --- | --- |
| VARIABLES | OLS I | OLS II | OLS III | OLS IV |
|  |  |  |  |  |
| UK | **0.201***** | **0.253***** | **0.232***** | **0.285***** |
|  | (0.044) | (0.050) | (0.045) | (0.051) |
| Post-IBI | -0.037 | 0.020 | -0.038 | 0.020 |
|  | (0.023) | (0.035) | (0.023) | (0.036) |
| UK x Post-IBI |  | **-0.105**** |  | **-0.106**** |
|  |  | (0.047) |  | (0.047) |
|  |  |  |  |  |
| Constant | -0.091** | -0.119*** | -0.489 | -0.518 |
|  | (0.037) | (0.040) | (0.327) | (0.327) |
|  |  |  |  |  |
| Observations | 3,261 | 3,261 | 3,237 | 3,237 |
| R-squared | 0.010 | 0.011 | 0.024 | 0.024 |
| Number of Observations | 3261 | 3261 | 3237 | 3237 |
| Dof | 2 | 3 | 10 | 11 |

**Notes:** Ordinary Least Squares (OLS) regressions, with safety as the dependent variables across. UK takes on a value of 0,1 (0=USA, 1=UK). Post-IBI takes on a value of 0, 1 (0 = Pre-IBI, 1= Post-IBI). Additional controls include age, gender, education, and prior donor status), clustered at the individual level to account for repeated observations over time. Cluster robust standard errors in parenthesis *** p < 0.01; ** p < 0.05; * p < 0.1

## Simple change index:

**Table S11: Summary statistics of simple change index (not RCI)**

|  | USA | UK | Total | p-value |
| --- | --- | --- | --- | --- |
| N(%) | 747 (45.7%) | 888 (54.3%) | 1,635 (100.0%) |  |
| Infection Risk |  |  |  |  |
| (-) Change | 158 (21.2%) | 163 (18.5%) | 321 (19.7%) | 0.144 |
| No Change | 471 (63.3%) | 555 (62.9%) | 1,026 (63.1%) |  |
| (+) Change | 115 (15.5%) | 164 (18.6%) | 279 (17.2%) |  |
| Safety |  |  |  |  |
| (-) Change | 211 (28.4%) | 307 (34.8%) | 518 (31.9%) | **<0.001***** |
| No Change | 285 (38.4%) | 361 (40.9%) | 646 (39.7%) |  |
| (+) Change | 247 (33.2%) | 215 (24.3%) | 462 (28.4%) |  |
|  |  |  |  |  |
| **Notes.** Summary statistics of the simple change index, with Chi-squared tests (for proportions). *** p < 0.01; ** p < 0.05; * p < 0.1. | | | | |

**Table S12: Logistic Regressions (in Odds Ratios) for Changes Across Pre- and Post-IBI (Simple change index)**

|  | **UK** | | **USA** | | **Total** | |
| --- | --- | --- | --- | --- | --- | --- |
| VARIABLES | **↓** $\Delta$ **Approach** | **↓** $\Delta$ **Encourage** | **↓** $\Delta$ **Approach** | **↓** $\Delta$ **Encourage** | **↓** $\Delta$ **Approach** | **↓** $\Delta$ **Encourage** |
|  |  |  |  |  |  |  |
| **Infection Risk:**  *Baseline: No change* |  |  |  |  |  |  |
| (-) | 0.749 | 1.004 | 0.812 | 0.865 | 0.784 | 0.927 |
|  | (0.178) | (0.216) | (0.207) | (0.209) | (0.134) | (0.147) |
| (+) | 0.824 | **0.652**** | 1.397 | 0.863 | 1.064 | **0.713**** |
|  | (0.189) | (0.138) | (0.380) | (0.238) | (0.179) | (0.118) |
|  |  |  |  |  |  |  |
| **Transfusion Safety:**  *Baseline: No change* |  |  |  |  |  |  |
| (-) | 1.304 | 1.380* | **1.753**** | **1.625**** | **1.458***** | **1.468***** |
|  | (0.249) | (0.241) | (0.406) | (0.359) | (0.212) | (0.201) |
| (+) | 1.270 | 0.828 | 0.956 | 1.099 | 1.073 | 0.924 |
|  | (0.283) | (0.176) | (0.231) | (0.254) | (0.175) | (0.142) |
|  |  |  |  |  |  |  |
| Constant | 1.512 | 0.409 | 0.590 | 0.271* | 0.840 | 0.251* |
|  | (1.035) | (0.328) | (0.438) | (0.212) | (0.555) | (0.201) |
|  |  |  |  |  |  |  |
| Region/State controls | ✓ | ✓ | ✓ | ✓ | ✓ | ✓ |
| Observations | 874 | 874 | 701 | 710 | 1,579 | 1,588 |
| Pseudo R2 | 0.0418 | 0.0330 | 0.0596 | 0.0520 | 0.0395 | 0.0357 |
| Log Likelihood | -457.3 | -512.8 | -364.1 | -385.1 | -831.4 | -905.6 |
| Degrees of Freedom | 24 | 24 | 49 | 50 | 62 | 63 |
| chi^2 | 37.64 | 33.88 | 47.82 | 44.77 | 67.36 | 65.71 |
| Prob < chi^2 | 0.0378 | 0.0867 | 0.521 | 0.682 | 0.299 | 0.383 |

**Notes:** Logistic regressions (represented by Odds Ratios, OR) for decreased changes in approach (↓ ∆ Approach) and encouraging others (↓ ∆ Encourage) by country (UK, USA). Independent variables represent simple changes (negative, no change, positive change) in values for (i) Infection Risk, and (ii) Transfusion Safety. Additional controls include age, gender, education, prior donor status and region/states residing in), clustered at the individual level to account for repeated observations over time. Cluster robust standard errors in parenthesis *** p < 0.01; ** p < 0.05; * p < 0.1

## Restricted sample:

**Table S13: Logistic Regressions (in Odds Ratios) for Changes Across Pre- and Post-IBI (restricted to first-week post-IBI)**

|  | **UK** | | **USA** | | **Total** | |
| --- | --- | --- | --- | --- | --- | --- |
| VARIABLES | **↓** $\Delta$ **Approach** | **↓** $\Delta$ **Encourage** | **↓** $\Delta$ **Approach** | **↓** $\Delta$ **Encourage** | **↓** $\Delta$ **Approach** | **↓** $\Delta$ **Encourage** |
|  |  |  |  |  |  |  |
| **Infection Risk:**  *Baseline: No change* |  |  |  |  |  |  |
| Sig. (-) | 0.649 | 0.999 | 0.613 | 0.660 | 0.648 | 0.801 |
|  | (0.368) | (0.476) | (0.342) | (0.368) | (0.248) | (0.283) |
| (-) | 0.779 | 0.985 | 0.912 | 0.917 | 0.833 | 0.956 |
|  | (0.208) | (0.240) | (0.274) | (0.252) | (0.164) | (0.173) |
| (+) | 0.887 | 0.683 | 1.429 | 0.770 | 1.109 | 0.711* |
|  | (0.228) | (0.164) | (0.445) | (0.262) | (0.211) | (0.137) |
| Sig. (+) | 0.658 | 0.445* | 1.361 | 1.101 | 0.947 | 0.688 |
|  | (0.326) | (0.202) | (0.720) | (0.558) | (0.324) | (0.220) |
|  |  |  |  |  |  |  |
| **Transfusion Safety:**  *Baseline: No change* |  |  |  |  |  |  |
| Sig. (-) | 1.518 | **2.581**** | **3.792***** | **2.180**** | **2.370***** | **2.370***** |
|  | (0.699) | (1.072) | (1.571) | (0.817) | (0.714) | (0.653) |
| (-) | 1.183 | 1.231 | **1.773**** | 1.580* | **1.372**** | **1.344**** |
|  | (0.240) | (0.229) | (0.457) | (0.386) | (0.216) | (0.198) |
| (+) | 1.254 | 0.847 | 0.912 | 1.054 | 1.045 | 0.903 |
|  | (0.295) | (0.189) | (0.250) | (0.270) | (0.186) | (0.150) |
| Sig. (+) | 0.859 | 0.317* | 1.523 | 0.711 | 1.089 | 0.462* |
|  | (0.466) | (0.203) | (0.814) | (0.445) | (0.407) | (0.203) |
|  |  |  |  |  |  |  |
| Constant | 1.514 | 0.478 | 0.568 | 0.338 | 0.788 | 0.283 |
|  | (1.044) | (0.388) | (0.430) | (0.269) | (0.542) | (0.233) |
|  |  |  |  |  |  |  |
| Region/State controls | ✓ | ✓ | ✓ | ✓ | ✓ | ✓ |
| Observations | 834 | 834 | 655 | 658 | 1,493 | 1,496 |
| Pseudo R2 | 0.0385 | 0.0409 | 0.0780 | 0.0565 | 0.0433 | 0.0405 |
| Log Likelihood | -433.8 | -483.5 | -330.5 | -355.7 | -775.6 | -847.6 |
| Degrees of Freedom | 28 | 28 | 53 | 53 | 66 | 66 |
| chi^2 | 33.29 | 40.43 | 55.28 | 44.49 | 68.26 | 69.15 |
| Prob < chi^2 | 0.225 | 0.0605 | 0.389 | 0.791 | 0.401 | 0.372 |

**Notes:** Logistic regressions (represented by Odds Ratios, OR) for decreased changes in approach (↓ ∆ Approach) and encouraging others (↓ ∆ Encourage) by country (UK, USA). Independent variables represent RCI values for (i) Infection Risk, and (ii) Transfusion Safety. Additional controls include age, gender, education, prior donor status and region/states residing in), clustered at the individual level to account for repeated observations over time. Sample restricted to those who answered within the first-week post-IBI (i.e., 30^th^ May to 5^th^ June 2024). Cluster robust standard errors in parenthesis *** p < 0.01; ** p < 0.05; * p < 0.1.

**Table S14: Logistic Regressions (in Odds Ratios) for Exploratory Demographics Analysis (restricted to first-week post-IBI)**

|  | **↑** $\boldsymbol{\Delta}$ **Risk** | | **↓** $\boldsymbol{\Delta}$ **Safety** | | **↓** $\boldsymbol{\Delta}$ **Approach** | | **↓** $\boldsymbol{\Delta}$ **Encourage** | |
| --- | --- | --- | --- | --- | --- | --- | --- | --- |
| VARIABLES | **USA** | **UK** | **USA** | **UK** | **USA** | **UK** | **USA** | **UK** |
|  |  |  |  |  |  |  |  |  |
| **Age** |  |  |  |  |  |  |  |  |
| *Baseline: Gen Z* |  |  |  |  |  |  |  |  |
| Millennials | **0.411**** | 0.676 | 0.924 | **2.197**** | 0.641 | 0.705 | 0.709 | 0.923 |
|  | (0.172) | (0.221) | (0.345) | (0.740) | (0.259) | (0.215) | (0.273) | (0.264) |
| Gen X + Boomers | 0.459* | 0.818 | 1.044 | **2.353**** | 0.869 | 0.644 | 0.975 | 0.809 |
|  | (0.189) | (0.269) | (0.393) | (0.800) | (0.346) | (0.200) | (0.368) | (0.233) |
| **Sex** |  |  |  |  |  |  |  |  |
| *Baseline: Males* |  |  |  |  |  |  |  |  |
| Female | 0.893 | **1.622***** | 1.008 | 1.268 | 0.766 | 1.020 | 0.951 | 0.745* |
|  | (0.207) | (0.300) | (0.186) | (0.189) | (0.151) | (0.172) | (0.180) | (0.115) |
| **Education** |  |  |  |  |  |  |  |  |
| *Baseline: Non-tertiary* |  |  |  |  |  |  |  |  |
| Tertiary | 0.761 | 1.128 | 1.202 | 1.039 | 0.789 | 1.030 | **1.645**** | 1.252 |
|  | (0.183) | (0.222) | (0.243) | (0.167) | (0.165) | (0.186) | (0.344) | (0.207) |
| **Donor history** |  |  |  |  |  |  |  |  |
| *Baseline: Non-donors* |  |  |  |  |  |  |  |  |
| Donor | 1.384 | 0.776 | 0.881 | 0.843 | 0.938 | **0.672**** | 1.114 | **0.663**** |
|  | (0.337) | (0.148) | (0.162) | (0.131) | (0.188) | (0.121) | (0.217) | (0.108) |
|  |  |  |  |  |  |  |  |  |
| Constant | 0.162 | 0.168*** | 0.167** | 0.258*** | 0.762 | 0.693 | 0.237* | 0.671 |
|  | (0.185) | (0.074) | (0.138) | (0.105) | (0.543) | (0.265) | (0.174) | (0.243) |
|  |  |  |  |  |  |  |  |  |
| Region/State controls | ✓ | ✓ | ✓ | ✓ | ✓ | ✓ | ✓ | ✓ |
| Observations | 622 | 834 | 665 | 834 | 659 | 834 | 662 | 834 |
| Pseudo R2 | 0.0637 | 0.0253 | 0.0396 | 0.0246 | 0.0440 | 0.0320 | 0.0411 | 0.0185 |
| Log Likelihood | -258.3 | -393.2 | -386.4 | -523.7 | -343.7 | -436.7 | -362.6 | -494.8 |
| Degrees of Freedom | 34 | 16 | 42s | 16 | 42 | 16 | 42 | 16 |
| chi^2 | 35.39 | 21.59 | 29.30 | 24.84 | 29.36 | 26.18 | 33.66 | 17.93 |
| Prob < chi^2 | 0.402 | 0.157 | 0.931 | 0.0727 | 0.930 | 0.0516 | 0.817 | 0.328 |

**Notes:** Logistic regressions (represented by Odds Ratios, OR) for increased changes in risk (↑ ∆ Risk), decreased changes in safety (↓ ∆ Safety), decreased changes in approach (↓ ∆ Approach) and encouraging others (↓ ∆ Encourage). Key demographics of interest are age (Gen Z, Millennials, Gen X + Boomers), sex (male, female), and prior donor status. Additional controls include education and region/states residing in). Standard errors are clustered at the individual level to account for repeated observations over time. Sample restricted to those who answered within the first-week post-IBI (i.e., 30^th^ May to 5^th^ June 2024). Cluster robust standard errors in parenthesis *** p < 0.01; ** p < 0.05; * p < 0.1
